# Influence of elevated temperature on the nutritional profile of Chickpea (*Cicer arietinum* L.) Seeds

**DOI:** 10.1371/journal.pone.0330230

**Published:** 2025-08-22

**Authors:** Uday Chand Jha, Marilyn Warburton, Harsh Nayyar, Sadiah Shafi, Ignacio A. Ciampitti, Ashis Ranjan Udgata, Kadambot H. M. Siddique, P. V. Vara Prasad

**Affiliations:** 1 Indian Council for Agricultural Research (ICAR) - Indian Institute of Pulses Research (IIPR), Kanpur, Uttar Pradesh, India; 2 Feed the Future Innovation Lab for Collaborative Research on Sustainable Intensification, Kansas State University, Manhattan, Kansas, United States of America; 3 USDA ARS, Western Regional Plant Introduction Station, Pullman, Washington, United States of America; 4 Department of Botany, Panjab University, Chandigarh, India; 5 Department of Agronomy, Kansas State University, Manhattan, Kansas, United States of America; 6 Department of Agronomy, Purdue University, West Lafayette, Indiana, United States of America; 7 The University of Western Australia – Institute of Agriculture, The University of Western Australia, Crawley, Perth, Australia; NABI: National Agri-Food Biotechnology Institute, INDIA

## Abstract

Increasing occurrences of episodic heat stress significantly affect crop quality traits, including those of chickpea (*Cicer arietinum* L.). The adverse effectof heat stress on seed quality was evaluated by cultivating eight chickpea genotypes under non-stress and heat stress conditions, with temperatures set at 25/15°C and 35/20°C, respectively. The genotypes exhibited notable genetic variations in *“seed carbon (C, %), protein (%), phosphorus (P, %), potassium (K, %), magnesium (Mg, %), sulfur (S, %),* and *manganese (Mn, ppm)”* concentrations under both conditions. However, no significant variations were observed for seed (S%), seed iron (Fe, ppm), and zinc (Zn, ppm), concentrations under NS conditions or seed copper (Cu, ppm) under heat stress conditions. The genotype (G) × temperature (T) interaction was significant for all traits except for seed K. Correlation analysis revealed positive associations between seed C and protein, seed Mg and P, and seed protein and S under non-stress (NS) conditions. Under heat stress, significant correlations were observed between seed protein and Mg, and seed protein and P. In contrast, significant negative correlations were observed between seed Ca and K under NS conditions and seed Ca and K and seed Fe and Cu under heat stress conditions. The adverse effects of heat stress on nutritional quality and seed yield underscore the necessity for continued research into developing heat-tolerant chickpea cultivars with enhanced seed nutritional traits.

## Introduction

Chickpea (*Cicer arietinum* L.) is a nutritionally rich pulse crop that serves as a vital source of bioavailable proteins, macronutrients, micronutrients, and vitamins, helping to alleviate malnutrition and ‘hidden hunger,’ particularly among low-income populations [1]. Chickpea contributes significantly to food security, with an annual global production of approximately 18.1 million tons (Mt) harvested from 14.8 million hectares (Mha) [2]. India is the leading producer, accounting for 11.08 Mt from 9.7 Mha annually [2]. As a cool-season legume, chickpea is well adapted to mild-temperature environments but highly susceptible to heat stress during vegetative and reproductive stages [3]. In northern and southern Indian regions, the increasing frequency of heat waves (>32°C) during the growing season, especially during the reproductive stage, has led to substantial yield losses [3–6] and has been attributed to climate change [7–9]. High-temperature stress at anthesis adversely affects pollen germination and viability, stigma receptivity, fertilization, and pod and seed development, ultimately reducing yield [3,6,10,11]. One study showed that exposure to high temperatures (30–35°C) during flowering reduces chickpea yield significantly [12]. Other studies reported thattemperatures exceeding 35°C reduced chickpea pod and seed set, decreasing yield by up to 39% [13,14]. Moreover, each additional degree above the optimal threshold (32°C) decreased chickpea yield by 15% [15] and increasing crop growing temperature by 4°C above ambient conditions reduced chickpea yield by 9–41% [16]. Beyond yield losses, heat stress also affects seed nutrient composition [17–19]. Chickpea seeds are rich in essential nutrients such as carbon (C), protein, phosphorus (P), magnesium (Mg), potassium (K), and sulfur (S), and micronutrients like iron (Fe), zinc (Zn), manganese (Mn), and copper (Cu).While extensive research has focused on the impact of heat stress on cereal grain quality [20], there is comparatively little informationon its effects on seed nutritional traits in grain legumes [19], including chickpea. In lentil, heat stress has been linked to an 8% reduction in starch content [21] and a decrease in seed protein concentration [22]. Similarly, heat stress decreased Zn, Fe, Ca, and Mg concentrations in lentil compared to non-stress conditions [23]. In chickpea, limited studies suggest a significant reduction in Fe and Zn concentrations under heat stress [24], with heat-sensitive genotypes showing Fe reductions of up to 59% and Ca reductions of 54% [18]. Given these findings, further investigation is necessary to understand how heat stress affects chickpea seed nutritional traits. This study aims to bridge this knowledge gap by evaluating the influence of elevated temperature on chickpea seed nutrient composition. The results will provide critical insights into developing heat-resilient chickpea varieties with improved nutritional quality.

## Materials and methods

### Plant materials

Eight diverse chickpea genotypes—PI 372596, PI 360688, PI 368485, PI 598080, PI 513144, PI 360691, PI 518255, and Gokce (see S1Table in S1 File)—were evaluated for seed yield and nutrient quality responses to heat stress. Seeds were sourced from the USDA ARS, Western Regional Plant Introduction Station, Pullman, Washington, USA. The experiment was conducted under NS conditions at Kansas State University, Manhattan, in 2024.

### Experimental set up

**Fig 1:**
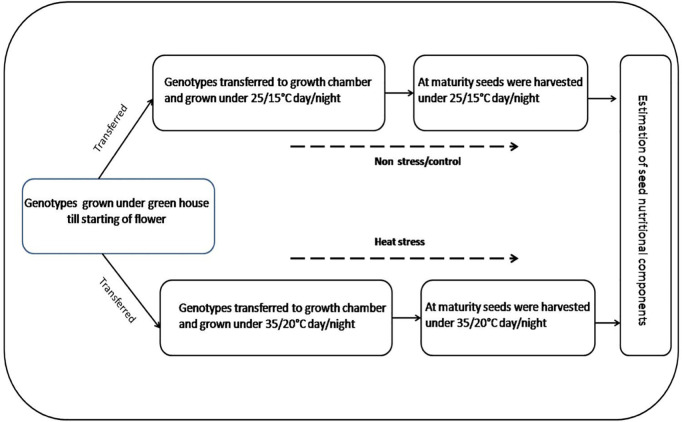
Graphical representation of methodology used in the experiment.

The study followed a randomized complete block design (RCBD) with three biological replicates, each comprising a single pot containing two plants. Seeds were sown on 29.1.2024 in 20 cm diameter pots filled with potting soil constituting (peat moss, vermiculite, compost, coir, garden soil, sand, manure, vermicompost and limestone) (Fafard^®^3B Mix/Metro-Mix^®^830, SUNGRO Horticulture, Agawam, MA, USA) “and maintained in a greenhouse for 60 days until flower initiation. At this stage, plants were transferred to a growth chamber last week of March, 28.3.2024) for controlled temperature treatments under non-stress conditions (NS; 25/15°C day/night) or heat stress conditions (HS; 35/20°C day/night) (see Fig.1). Plants were exposed to photosynthetically active radiation (400–700 nm) of 600 μmol m^–2^ s^–1^under a 12 h photoperiod provided by cool fluorescent lamps. The relative humidity was maintained at 60%, and plants were watered regularly to prevent drought stress. A consistent nutrient supply was maintained (for details, see Jha et al. [25]). Temperature data were recorded using a HOBO^®^data logger (Onset Computer Corporation, Bourne, Massachusetts, USA)”(S1 Fig in S1 File). Plants were harvested at physiological maturity for seed yield and nutritional quality analysis.

### Analysis of seed nutritional components

Seed samples (0.5 g per genotype) were collected in triplicate and analyzed for various nutritional components: protein (%), C (%), P (%), K (%), Mg (%), Ca (%), S (%), Mn (ppm), Cu (ppm), Fe (ppm), and Zn (ppm). All analyses were performed at the Kansas State Soil Testing Laboratory (Manhattan, KS, USA). Total carbon (C) and nitrogen (N) concentrations were determined using a LECO TruSpec CN Carbon/Nitrogen combustion analyzer (LECO Corporation, St. Louis, MO, USA). Concentrations of P, K, Mg, Ca, S, Mn, Cu, Fe, and Zn were measured using a nitric-perchloric digest method, followed by analysis with an Inductively Coupled Plasma (ICP) spectrometer (Model 5800 ICP OES, Agilent Technologies, Santa Clara, California, USA) [26,25]. Seed protein concentration was calculated using the Kjeldahl method, multiplying N concentration by 6.25 [27]. The entire nutrient estimation process followed the methodology outlined by Jha et al. [25].

### Seed yield per plant (SYP)

At maturity, plants were harvested, and seed yield per plant (SYP) was determined by threshing the seeds from two plants per replicate and calculating the average yield.

### Data analyses

Analysis of variance (ANOVA) was conducted using OPSTAT software, with the least significant difference (LSD) calculated at the 5% and 1% significance levels. Correlation analyses for seed nutrient traits and seed yield per plant (SYP), principal component analysis (PCA), and genotype clustering were all performed using RStudio.

## Results

Under NS, ANOVA revealed significant genetic variation among the eight chickpea genotypes for all nutrient parameters except seed iron (Fe), zinc (Zn), and copper (Cu) concentrations. The observed ranges under NS condition were: protein (14.2–22.9%), carbon (C) (40.4–41.2%), phosphorus (P) (0.38–0.55%), potassium (K) (1.21–1.58%), calcium (Ca) (0.07–0.17%), magnesium (Mg) (0.15–0.19%), sulfur (S) (0.21–0.25%), manganese (Mn) (25.2–45.7 ppm), zinc (Zn) (67.7–83.6 ppm), copper (Cu) (2.13–4.96 ppm), iron (Fe) (58.8–101.2 ppm), and seed yield per plant (SYP) (4.1–8.77 g) (Tables 1 and 2). Conversely, under high salinity (HS) conditions, significant genetic variation was observed for all traits except seed Fe concentration, with the following ranges: protein (16.3–26.4%), C (40.1–41.18%), P (0.44–0.65%), K (1.29–1.56%), Ca (0.09–0.16%), Mg (0.16–0.20%), S (0.24–0.30%), Mn (26.9–52.8 ppm), Zn (67.9–91.4 ppm), Cu (1.73–3.20 ppm), Fe (41.5–81.3 ppm), and SYP (2.6–4.9 g) (Tables 1 and 3). Furthermore, a combined ANOVA indicated significant genotype × treatment (G × T) interactions for all traits except seed K concentration (Table 4 and S2Table in S1 File).

**Table 1:** Genetic variability for chickpea seed nutrient components under non-stress (control) and heat stress conditions.

	Treatment	Protein (%)	C (%)	P (%)	K (%)	Ca (%)	Mg (%)	S (%)	Mn (ppm)	Zn (ppm)	Cu (ppm)	Fe (ppm)	SYP (g)
Minimum	NS	14.2	40.42	0.38	1.21	0.07	0.15	0.21	25.2	67.67	2.13	58.8	4.1
	HS	16.3	40.12	0.44	1.29	0.09	0.16	0.24	26.9	67.9	1.73	41.53	2.6
Maximum	NS	22.9	41.22	0.55	1.58	0.17	0.19	0.25	45.7	83.63	4.96	101.2	8.77
	HS	26.4	41.18	0.65	1.56	0.16	0.2	0.3	52.8	91.43	3.2	81.3	4.9
Mean	NS	18.38	40.88	0.48	1.36	0.121	0.170	0.23	32.98	74.93	2.86	74.5	6.76
	HS	19.34	40.62	0.54	1.44	0.116	0.178	0.27	35.02	75.68	2.37	63.42	3.39
Standard error	NS	1.21	0.11	0.02	0.041	0.014	0.005	0.005	2.52	2.25	0.32	5.98	0.58
	HS	1.28	0.14	0.021	0.04	0.010	0.005	0.008	3.1	3.12	0.17	4.66	0.26
Coefficient of variation	NS	18.60	0.76	11.6	8.64	33.1	8.3	6.3	21.5	8.5	31.7	22.7	24.3
	HS	18.70	0.95	11.1	7.6	23.8	7.2	8.5	25.3	11.7	20.6	20.7	22.1
NS: non-stress; HS:heat stress SYP: seed yield per plant													

**Table 2:** Seed nutritional and seed yield per plant (SYP) for eight chickpea genotypes under non-stress conditions.

Genotype	Protein (%)	Carbon (%)	Phosphorus (%)	Potassium (%)	Calcium (%)	Magnesium (%)	Sulfate (%)	Copper (ppm)	Iron (ppm)	Manganese (ppm)	Zinc (ppm)	SYP (g)
PI368485	22 ± 0.56^a^	41.223 ± 0.07^a^	0.501 ± 0.01^ab^	1.39 ± 0.03^ab^	0.066 ± 0.002^c^	0.162 ± 0.002^b^	0.226 ± 0.003	2.367 ± 0.75^b^	58.833 ± 2.5	26.5 ± 0.8^c^	70.267 ± 3.2	7.57 ± 0.23^b^
PI518255	17 ± 1.8^bc^	40.733 ± 0.1^b^	0.498 ± 0.01^ab^	1.30 ± 0.04^b^	0.162 ± 0.003^a^	0.165 ± 0.002^b^	0.228 ± 0.004	2.567 ± 0.15^b^	65.333 ± 1.58	45.667 ± 1.1^a^	76.133 ± 3.5	8.7 ± 0.18^a^
PI360691	21.967 ± 1.4^a^	41.11 ± 0.13^a^	0.505 ± 0.01^ab^	1.39 ± 0.02^ab^	0.102 ± 0.01^b^	0.192 ± 0.01^a^	0.253 ± 0.01	3.2 ± 0.6^b^	80.833 ± 22.7	26.433 ± 2.6^c^	69.633 ± 1.2	4.1 ± 0.15^d^
PI598080	22.9 ± 1.36^a^	40.69 ± 0.12^b^	0.483 ± 0.01^b^	1.36 ± 0.07^b^	0.151 ± 0.02^a^	0.169 ± 0.01^b^	0.25 ± 0.02	2.767 ± 0.03^b^	101.167 ± 2.57	38.3 ± 1.8^ab^	83.033 ± 5.2	7.8 ± 0.12^b^
PI360688	17.833 ± 0.3^b^	41.123 ± 0.1^a^	0.408 ± 0.01^c^	1.4 ± 0.01^ab^	0.088 ± 0.01^bc^	0.145 ± 0.01^c^	0.222 ± 0.01	2.133 ± 0.2^b^	97.7 ± 15.1	33b ± 3.4^c^	78.867 ± 2.7	5.16 ± 0.01^c^
Gocke	15.733 ± 1.24^bc^	41.16 ± 0.2^a^	0.382 ± 0.04^c^	1.22 ± 0.16^b^	0.153 ± 0.02^a^	0.159 ± 0.01^bc^	0.222 ± 0.02	4.967 ± 0.5^a^	63.167 ± 14.7	25.167 ± 5.3^c^	67.667 ± 17.7	5.3 ± 0.01^c^
PI513144	15.433 ± 0.12^bc^	40.42 ± 0.05^c^	0.547 ± 0.01^a^	1.57 ± 0.01^a^	0.077 ± 0.01^bc^	0.189 ± 0.001^a^	0.235 ± 0.002	2.467 ± 0.2^b^	59.233 ± 2.5	31.6b ± 1.4^c^	83.633 ± 1.9	7.5 ± 0.01^b^
PI372596	14.2 ± 0.7^c^	40.547 ± 0.05^bc^	0.468 ± 0.002^b^	1.21 ± 0.03^b^	0.168 ± 0.01^a^	0.173 ± 0.004^b^	0.208 ± 0.01	2.4 ± 0.8^b^	69.767 ± 4.4	37.133 ± 2.6^ab^	70.167 ± 5.2	7.8 ± 0.01^b^

Data are mean ± standard error of the mean. Different lowercase letters within a column indicate significant differences at *P* < 0.05 as per Tukey’s honest significant difference (HSD)test.

**Table 3:** Seed nutritional profile and seed yield per plant (SYP) for eight chickpea genotypes under heat stress conditions.

Genotype	Protein (%)	Carbon (%)	Phosphorus (%)	Potassium (%)	Calcium (%)	Magnesium (%)	Sulfate (%)	Copper (ppm)	Iron (ppm)	Manganese (ppm)	Zinc (ppm)	SYP (g)
PI368485	26.4 ± 2.6^a^	40.583 ± 0.1^bc^	0.649 ± 0.01^a^	1.55 ± 0.03^a^	0.092 ± 0.003^d^	0.203 ± 0.003^a^	0.299 ± 0.001^a^	2.233 ± 0.5	68.43 ± 4.4^ab^	37.47 ± 2.45^b^	91.43 ± 0.93^a^	2.5 ± 0.01^f^
PI518255	17.1 ± 0.86^c^	40.19 ± 0.1^bc^	0.545 ± 0.01^b^	1.41 ± 0.06^abc^	0.141 ± 0.003^ab^	0.174 ± 0.00^cd^	0.286 ± 0.004^ab^	2.5 ± 0.5	52.66 ± 3.1^bc^	41.06 ± 1.4^b^	69.4 ± 2^c^	4.9 ± 0.12^a^
PI360691	22.83 ± 0.2^ab^	40.44 ± 0.2^bc^	0.556 ± 0.02^b^	1.29 ± 0.04^c^	0.123 ± 0.01^bc^	0.186 ± 0.004^b^	0.259 ± 0.01^bc^	3.2 ± 0.2	41.53 ± 1.7^c^	27.23 ± 3^d^	69.167 ± 2.4^c^	2.9 ± 0.12^e^
PI598080	16.4 ± 0.2^c^	40.58 ± 0.1^bc^	0.49 ± 0.01^bd^	1.36 ± 0.03^bc^	0.164 ± 0.002^a^	0.168 ± 0.004^d^	0.239 ± 0.01^c^	2.567 ± 0.1	58.13 ± 1.6^c^	52.76 ± 2.5^a^	67.9 ± 2.4^c^	3.5 ± 0.06^c^
PI360688	16.3 ± 1.38^c^	41.18 ± 0.2^a^	0.442 ± 0.004^d^	1.51 ± 0.06^ab^	0.093 ± 0.01^d^	0.155 ± 0.01^e^	0.247 ± 0.004^c^	1.833 ± 0.3	68.43 ± 0.5^ab^	26.9 ± 3.2^d^	69.03 ± 1.6^c^	2.7 ± 0.06e^f^
Gocke	18.9 ± 0.8^c^	41.123 ± 0.2^a^	0.548 ± 0.02^b^	1.51 ± 0.03^ab^	0.104 ± 0.003^cd^	0.183 ± 0.004^bc^	0.28 ± 0.01^ab^	1.733 ± 0.3	77.73 ± 1.6^a^	30.43 ± 0.8^cd^	84.3 ± 2.7^b^	3.39 ± 0.06^cd^
PI513144	19.83 ± 0.9^bc^	40.72 ± 0.3^ab^	0.537 ± 0.02^bc^	1.55 ± 0.04^a^	0.085 ± 0.004^d^	0.17 ± 0.003^d^	0.296 ± 0.01^a^	2.767 ± 0.2	59.1 ± 1.6^b^	35.83 ± 2.3^bc^	81.26 ± 3.5^b^	3.9 ± 0.06^b^
PI372596	16.9 ± 0.9^c^	40.12 ± 0.04^c^	0.547 ± 0.02^b^	1.31 ± 0.1^c^	0.137 ± 0.02^ab^	0.184 ± 0.002^b^	0.259 ± 0.02^bc^	2.133 ± 0.6	81.3 ± 13.7^a^	28.53 ± 1.8^d^	72.9 ± 1.4^c^	3.2 ± 0.2^d^

Data are mean ± standard error of the mean. Different lowercase letters within a column indicate significant differences at *P* < 0.05 as per Tukey’s honest significant difference (HSD)test.

**Table 4:** Significance of temperature (T), genotype (G), and T × G interactions on seed nutritional profile and seed yield per plant (SYP).

	Variables			Mean	
**Trait**	**Treatment(T)**	**Genotype(G)**	**T × G**	**Control**	**Heat stress**
Protein (%)	10.92	53.9**	20.2**	18.4^a^	19.3^a^
Carbon (%)	0.77	0.54**	0.2**	40.8^a^	40.6^b^
Phosphorus (%)	0.051**	0.013**	0.006**	0.47^a^	0.54^b^
Potassium(%)	0.077*	0.054 **	**0.024**	1.36^a^	1.44^a^
Calcium(%)	0	0.007**	0.001**	0.121^a^	0.117^b^
Magnesium(%)	0.001**	0.001**	0**	0.169^a^	0.178^a^
Sulfur(%)	0.019**	0.001*	0.001**	0.230^a^	0.271^b^
Manganese (ppm)	50.6	284.96**	101.6**	32.9^a^	35.01^a^
Zinc (ppm)	6.93	119.49	236.73*	74.9^a^	75.6^a^
Copper (ppm)	2.85**	1.3	1.9*	2.85^a^	2.37^a^
Iron (ppm)	1,475.3*	550.8	828.6**	74.5^a^	63.4^a^
SYP(g)	113.02**	5.79**	1.73**	4.524^a^	1.779^b^

Means were separated using Tukey’s honest significant difference (HSD) test at *P* = s0.05. Different superscripted letters and numbers in bold indicate significant effects on variables or means.

### Protein and carbon concentration

**Fig 2:**
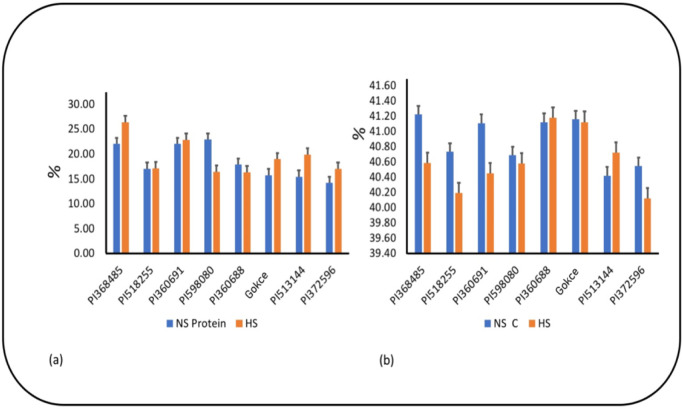
(a) Seed protein concentration of chickpea genotypes under non-stress(NS) and heat stress (HS)conditions. (b) Seed carbon (C) concentration of chickpea genotypes under non-stress(NS) and heat stress (HS)conditions Values are means + SE. (*n *= 3). Tukey’s test was used to examine treatment differences (*P* < 0.05).

The average protein concentration increased from 18.38% to 19.34% under HS (Fig 2), whereas the carbon concentration slightly decreased from 40.88% to 40.62%. The protein concentration exhibited significant genotype and G × T interaction effects (Table 4). Deviating from the trend observed in the other genotypes under HS, PI598080 and PI360688 exhibited a decrease in protein concentrations, whereas PI360688 and PI513144 exhibited an increase in seed C (see Table 3).

### Primary and secondary macronutrients

**Fig 3:**
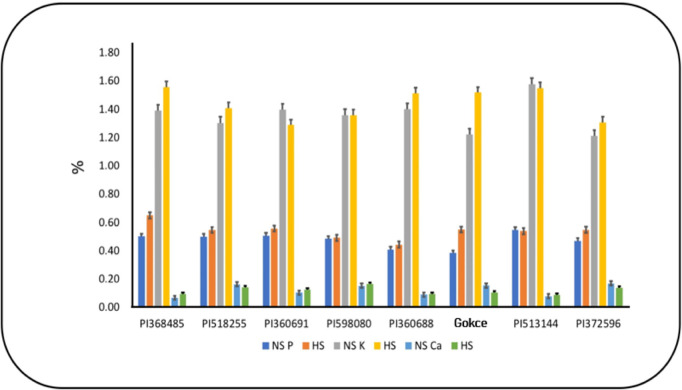
(a) Seed phosphorus (P),potassium (K), and calcium(Ca) concentrations of chickpea genotypes under non-stress(NS) and heat stress (HS)conditions. Values are means + SE (*n *= 3). Tukey’s test was used to examine treatment differences (*P* < 0.05).

**Fig 4:**
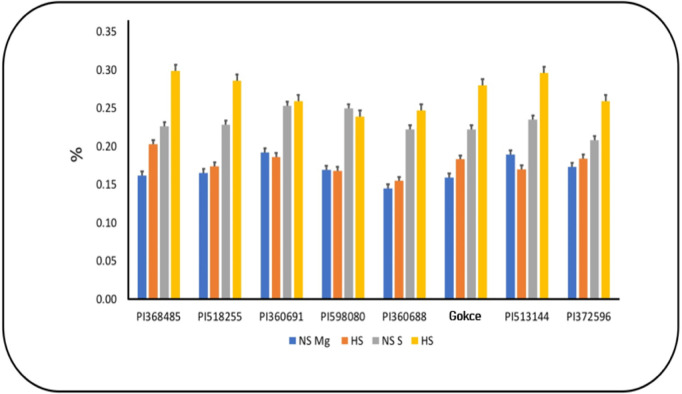
Seed magnesium (Mg) and sulfur (S) concentrations for chickpea genotypes under non-stress(NS) and (b) heat stress (HS) conditions. Values are means + SE (**n* *= 3). Tukey’s test was used to examine treatment differences (*P* < 0.05).

Total seed K had individual G and T effects but no significant G × T interaction (Table 4 and S2 Table in S1 File). Seed P, Mg, and S concentrations exhibited significant T, G, and G × T interaction effects, typically increasing under HS (Figs 3 and 4). However, PI513144 and PI598080 decreased Mg and S concentrations under HS, respectively. Seed Ca had significant treatment and interaction effects, typically decreasing under HS (Fig 3). However, PI 368485 and PI 360691 increased Ca concentration under HS.

### Micronutrients

**Fig 5:**
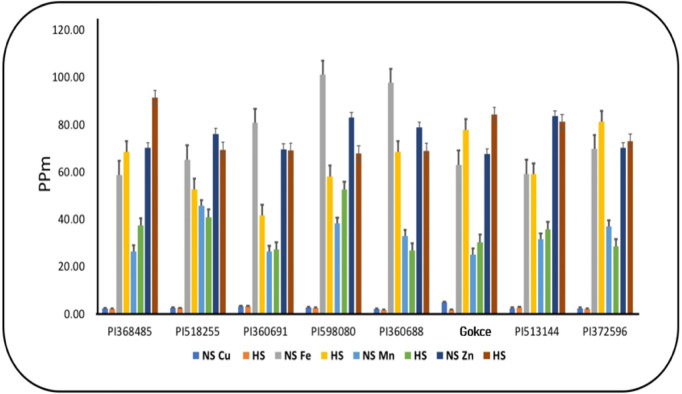
Seed copper (Cu), iron (Fe), manganese (Mn), and zinc (Zn) concentrations for chickpea genotypes under non-stress(NS) and heat stress (HS) conditions. Values are means + SE (**n* *= 3). Tukey’s test was used to examine treatment differences (*P* < 0.05).

Seed Cu, Zn, Fe, and Mn concentrations displayed significant genotype (G) × temperature (T) interaction effects (Table 4), with seed Zn and Fe also showing significant temperature effects. Under heat stress (HS), seed Zn and Mn concentrations increased, while seed Fe and Cu concentrations typically decreased (Fig 5). However, counter to the general trend, PI 513144 increased seed Cu concentration, and Gocke, PI 368485, and PI 372596 increased Fe concentrations under HS (Table 3 and Fig 5).

### Seed yield per plant

**Fig 6:**
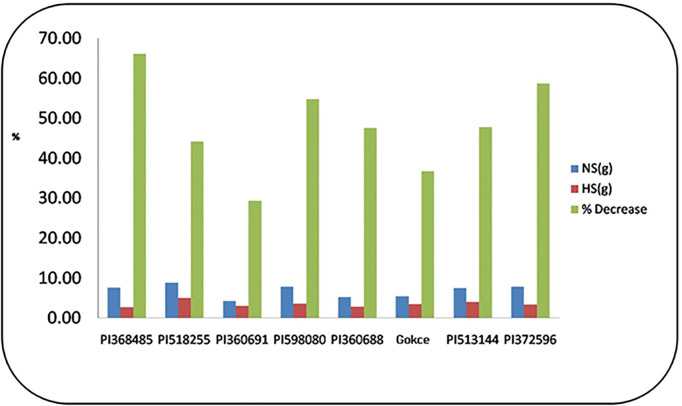
Percentage reduction in seed yield/plant (g) for eight selected chickpea genotypes under heat stress. NS = non-stress, HS = heat stress.

We observed a significant decrease in seed yield per plant (SYP) (29–66%, Fig 6) due to heat stress, with strong influence from genotype (G), temperature (T), and their interaction (G × T). Notably, PI360691 demonstrated the lowest SYP reduction under heat stress among all tested genotypes, identifying it as a potential candidate for heat tolerance breeding.

### Correlation analysis

**Fig 7:**
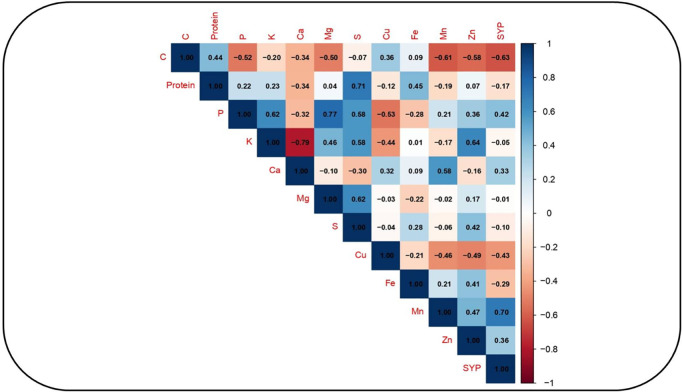
Correlation analysis forvarious nutritional traits in eight selected chickpea genotypes under non-stress conditions. Protein = seed protein (%); C = seed carbon (%), P = phosphorous (%), K = potassium (%), Ca = calcium (%), Mg = magnesium (%), S = sulfur (%), Mn = manganese (ppm), Zn = zinc (ppm), Cu = copper (ppm), Fe = iron (ppm), and SYP = seed yield per plant **(g)**.

Under NS, significant positive association occurred between seed C and protein content (0.44), seed Mg and P content (0.77), and seed S and protein content (0.71) (Fig 7). Seed Ca negatively correlated with K (–0.79).

**Fig 8:**
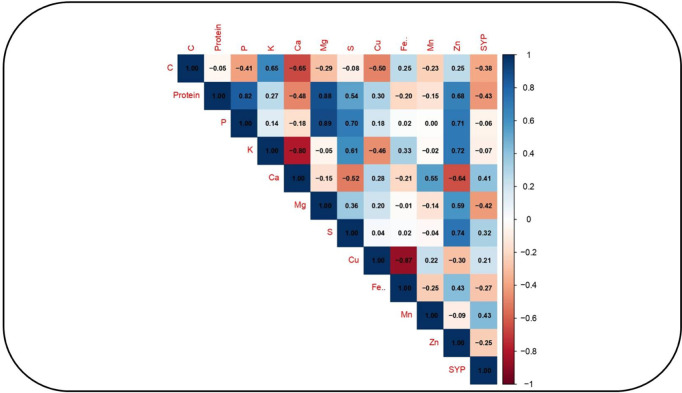
Correlation analysis forvarious nutritional traits in eight selected chickpea genotypes under heat stress conditions. Protein = seed protein (%); C = seed carbon (%), P = phosphorous (%), K = potassium (%), Ca = calcium (%), Mg = magnesium (%), S = sulfur (%), Mn = manganese (ppm), Zn = zinc (ppm), Cu = copper (ppm), Fe = iron (ppm), and SYP = seed yield per plant **(g)**.

Under HS, positive association occurred between seed protein and Mg (0.88), seed protein and P (0.82), seed Mg and P (0.89), seed Zn and P (0.71), seed Zn and K (0.72), and seed Zn and S (0.74) (Fig 8), whereas negative association occurred between seed Ca and K (–0.80) and seed Fe and Cu (–0.87).

### Principal component (PC) analysis

**Fig 9:**
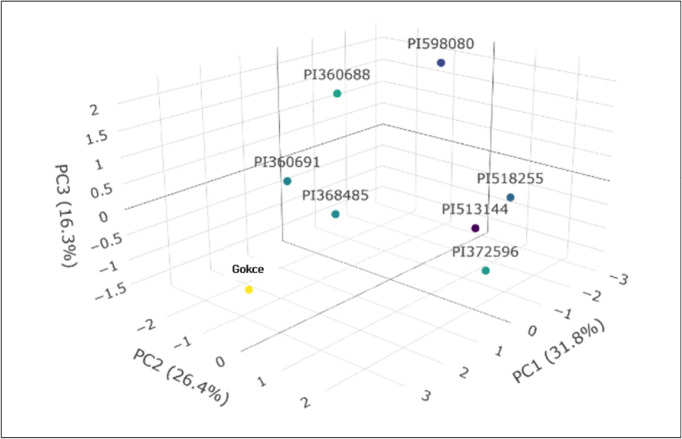
Principal component analysis (PCA) for various seed nutritional traits in eight selected chickpea genotypes under non-stress conditions.

Analysis under non-stress (NS) conditions revealed that three PCs collectively accounted for 74.5% of the total variability across the 11 traits (Figs 9 and 11). Individually, PC1 contributed 31.8%, PC2 26.4%, and PC3 16.3%.

**Fig 10:**
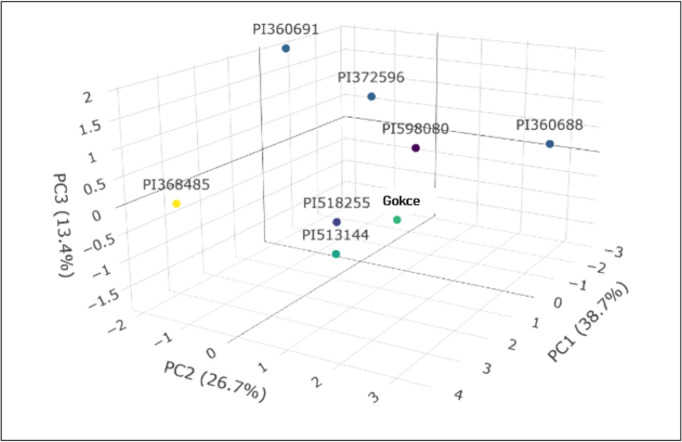
Principal component analysis (PCA) for various seed nutritional traits in eight selected chickpea genotypes under heat stress conditions.

Examining the component loadings (Table 5), PC1 was positively driven by P (0.76), K (0.80), Zn (0.63), and S (0.64), with C (–0.64) exhibiting the most pronounced negative contribution. PC2 showed C (0.65) and protein (0.63) as the strongest positive contributors, while Ca (–0.75) was the most negatively influential. For PC3, Fe (0.91) and protein (0.46) were the primary positive contributors, and Mg (−0.52) had the most significant negative impact. PC4 saw Ca (0.56) as its largest positive contributor, contrasted by K (–0.31) as the most significant negative contributor.

**Table 5:** Correlation between variables and PCs under non-stress conditions.

Variables	PC1	PC2	PC3	PC4	PC5	PC6	PC7
Carbon (%)	–0.64	0.65	0.17	–0.13	0.281	0.042	0.203
Protein (%)	0.23	0.63	0.46	0.27	0.501	0.072	–0.09
Phosphorus (%)	0.9	0.05	–0.35	0.07	0.28	–0.139	0.008
Potassium (%)	0.8	0.48	–0.1	–0.31	–0.246	0.122	0.069
Calcium (%)	–0.32	–0.75	0.13	0.56	0.048	–0.028	–0.017
Magnesium (%)	0.63	0.15	–0.52	0.49	–0.148	–0.236	0.014
Sulfur (%)	0.64	0.53	0.12	0.52	0.063	0.158	0.064
Copper(ppm)	–0.64	0.17	–0.29	0.52	–0.232	0.38	0.032
Iron (ppm)	0.07	0.16	0.91	0.19	–0.25	–0.213	–0.094
Manganese(ppm)	0.37	–0.79	0.37	0.102	0.109	–0.05	0.291
Zinc (ppm)	0.76	–0.14	0.41	–0.13	–0.383	0.291	0.003
Seed yield per plant(g)	0.43	–0.72	–0.03	–0.11	0.409	0.312	–0.113

**Fig 11:**
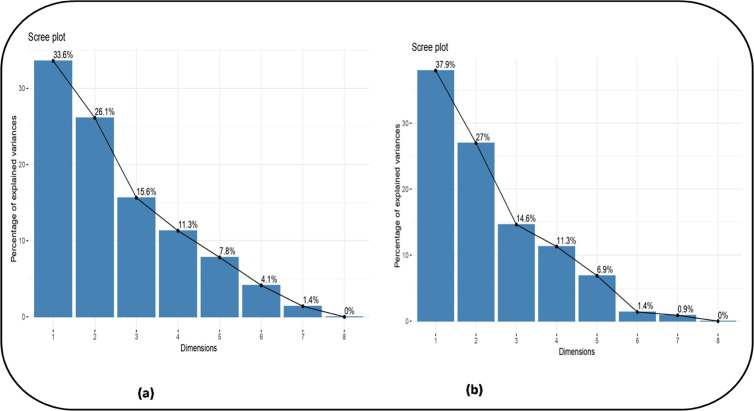
Scree plot constructed using eightprincipal components for eight selected chickpea genotypes under (a) non-stress and (b) heat stress conditions.

Under HS, the initial four principal components (PCs) collectively accounted for 78.8% of the total variability. PC1 contributed 38.7%, PC2 26.7%, and PC3 13.4% (Figs 10 and 11). PC1 was primarily driven by positive contributions from Zn (0.95), protein (0.79), P (0.70), and Mg (0.64), with Ca showing the most significant negative influence (–0.79). For PC2, Cu (0.79) and P (0.65) were the strongest positive contributors, while C exhibited the most notable negative contribution (–0.78) (Table 6). SYP (0.82) and S (0.58) were the main positive drivers for PC3, and Mg had the greatest negative impact (–0.37). Finally, PC4 saw Fe (0.71) as its largest positive contributor, with K showing the greatest negative contribution (0.21).

**Table 6:** Correlation between variables and PCs under heat stress conditions.

Variables	PC1	PC2	PC3	PC4	PC5	PC6	PC7
Carbon (%)	0.3	–0.78	–0.04	–0.4	0.259	0.226	0.122
Protein (%)	0.79	0.52	–0.23	–0.19	0.144	0.009	–0.074
Phosphorus (%)	0.701	0.65	0.05	0.27	–0.034	0.068	–0.02
Potassium (%)	0.7	–0.47	0.47	–0.21	0.149	–0.081	–0.086
Calcium (%)	–0.79	0.39	0.02	0.43	0.168	0.092	0.03
Magnesium (%)	0.64	0.6	–0.37	0.22	0.104	0.145	0.014
Sulfur (%)	0.71	0.27	0.58	–0.05	–0.281	–0.028	–0.014
Copper (ppm)	–0.25	0.79	–0.05	–0.5	–0.009	–0.159	0.193
Iron (ppm)	0.32	–0.6	–0.04	0.71	–0.054	–0.127	0.109
Manganese (ppm)	–0.32	0.29	0.54	0.12	0.703	–0.059	–0.024
Zinc (ppm)	0.95	0.005	0.19	0.17	0.128	–0.019	0.154
Seed yield per plant (g)	–0.4	0.23	0.82	0.04	–0.288	0.169	0.049

### Clustering analysis

**Fig 12:**
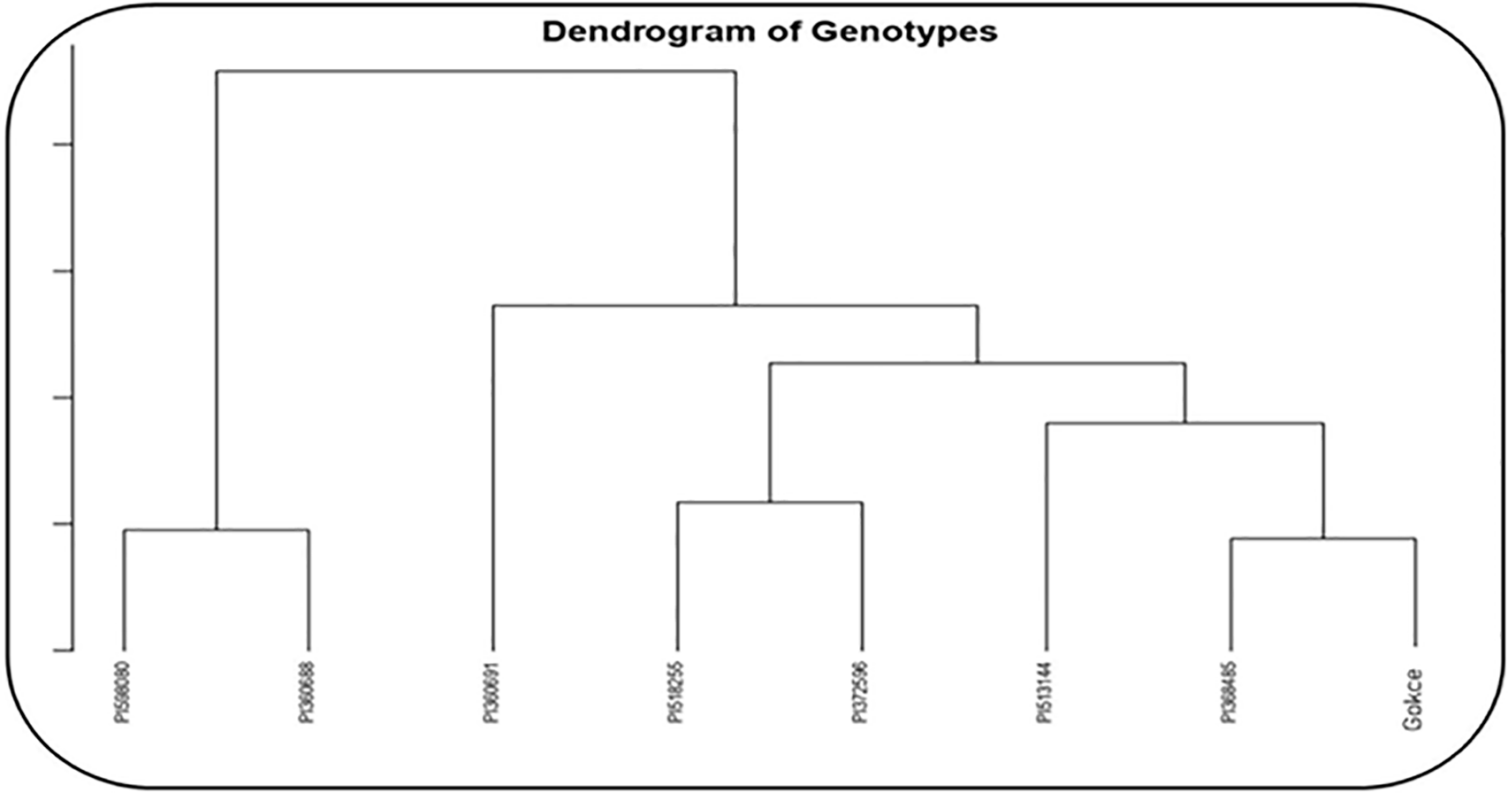
Cluster analysis of eight selected chickpea genotypes under non-stress conditions.

**Fig 13:**
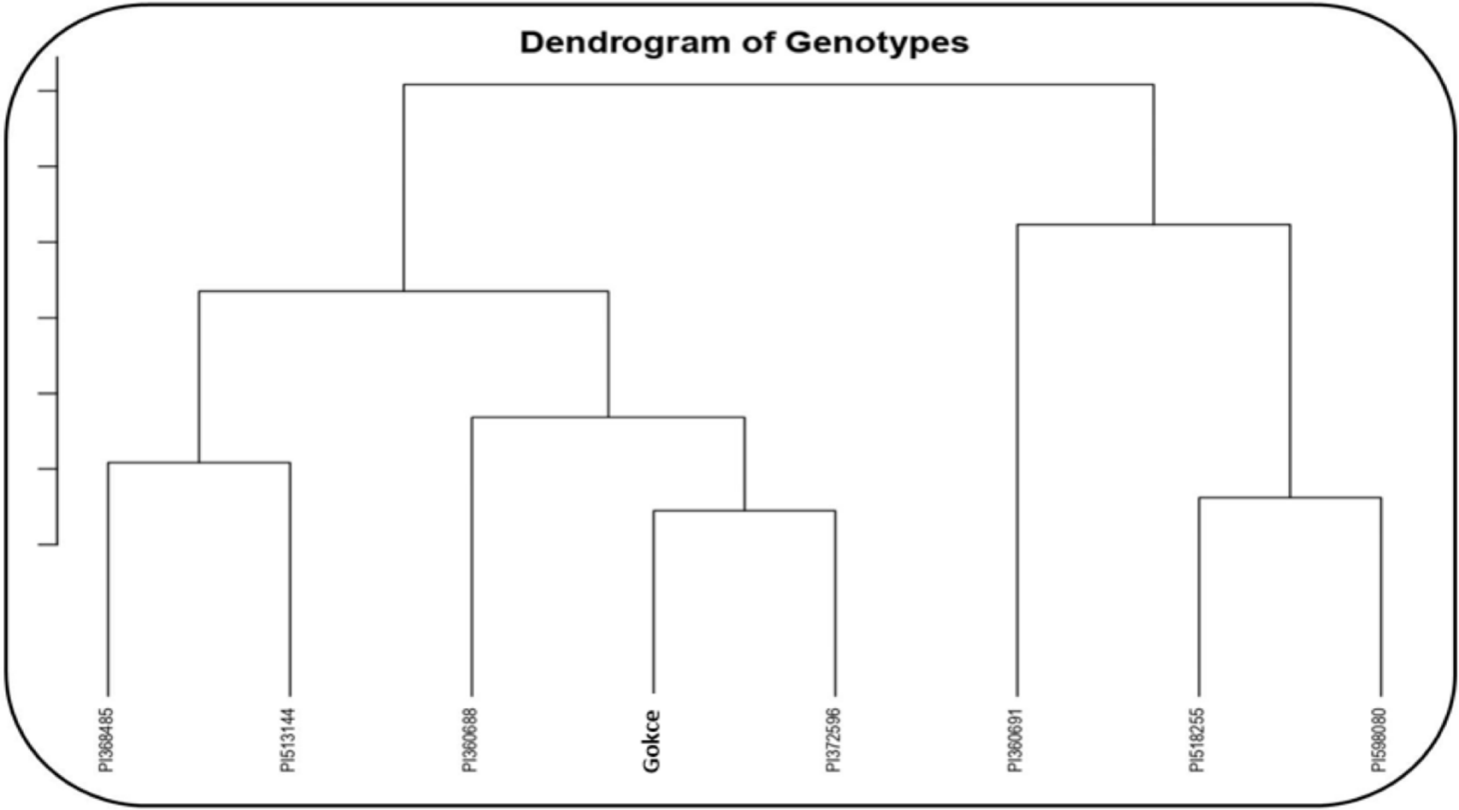
Cluster analysis of eight selected chickpea genotypes under heat stress conditions.

Under NS, the genotypes formed two main clusters: (1) Gocke, PI368485, PI513144, PI518255, PI372596, and PI360691 and (2) PI598080 and PI360688 (Fig 12). Under HS, the genotypes also grouped into two clusters: (1) PI360688, Gokce, PI372596, PI513144, and PI368485 and (2) PI360691, PI518255, and PI598080 (Fig 13).

## Discussion

Heat stress significantly affects crop yields and is exacerbated by significant genotype × temperature (G × T) interactions [6,11]. Heat stress also alters the seed macronutrient and micronutrient components in various grain legumes, including chickpea [18,22,28,29].

Our study revealed that heat stress in chickpea decreased seed C concentration but increased seed protein concentration. Similar reductions in seed carbohydrates due to HS have been reported in maize (*Zea mays* L.), with declines of 3.0% in 2014 and 3.3% in 2015 [30], and in rice (*Oryza sativa* L.), where HS disrupted sucrose synthase and starch branching enzyme activities, hindering starch accumulation [31]. In wheat (*Triticum aestivum* L.), HS inhibits key enzymes involved in starch metabolism, such as soluble starch synthase, sucrose synthase, glucokinase, and ADP glucose pyrophosphorylase, reducing carbohydrate levels [32]. Similar trends have been reported in grain legumes such as chickpea [18,21,23,29]. The decline in carbohydrate concentration is likely due to reduced enzymatic activity in starch and sucrose synthesis, including sucrose phosphate synthase, soluble starch synthase, and sucrose synthase [18,30], coupled with restricted sucrose transport from leaves to developing pods and seeds [31].

In contrast to carbohydrate depletion, seed protein concentration often increases under HS, as reported in wheat [33,34], maize (24.5% in 2014 and 25.3% in 2015) [30], hard fescue (*Festuca trachyphylla*) [35], and soybean (*Glycine max* L.) [36]. This increase is attributed to enhanced enzyme activity, including “*glutamate synthase and glutamate pyruvate transaminase*”, along with decreased glutamine synthetase activity during grain filling [30]. Additionally, increased cellular production of heat shock proteins, heat shock factors, and stress signaling proteins, which help plants adapt to HS, contribute to this increase in protein [30,37]. However, HS has been reported to decrease seed protein concentrations in crops such as *Vigna radiata* [29,37], *Cicer arietinum* [18,24,38], soybean [37], *Lens culinaris* [22], and *Lathyrus sativus* L [28].

Our study also found significant T, G, and G × T interaction effects for seed P, Mg, Ca, and S concentrations, which increased under HS. Phosphorus is critical for mitigating HS by influencing seed metabolism, reducing seed oil and starch concentrations [39], and enhancing seed quality traits; it is also essential in mediating mitogen-activated protein kinase cascades during HS [40]. Magnesium, vital for chlorophyll synthesis and carbon metabolism, typically increases under heat stress, aiding plant tolerance [41]. However, decreased seed Mg, Ca, and K concentrations have been noted in mung bean [29] and lentil [21] under similar conditions. In this study, seed Ca concentration decreased under HS, likely due to its role as an intracellular signaling molecule in HS responses [42]. In contrast, seed S concentration increased, which may be linked to its role in synthesizing methionine—an amino acid crucial for heat shock protein function [43]—and its involvement in maintaining cellular redox balance and scavenging reactive oxygen species (ROS) [44,45]. Notably, exogenous S application alleviated heat stress in tomato plants by increasing proline, N, P, and K contents [46].

Micronutrient concentrations also shifted under HS, with a decline in seed Fe levels, consistent with previous reports in chickpea [17,18,24], lentil [21–23,47], and mung bean [29]. Conversely, seed Zn concentration increased, which may enhance C assimilation and alleviate oxidative stress through antioxidant production [48]. Similarly, Mn levels increased in chickpea under HS [17], potentially contributing to improved ROS scavenging and upregulation of chaperone proteins, enhancing HS tolerance [49,50]. However, reductions in seed Zn concentration under HS have also been reported in chickpea [17,24], lentil [21,22], and mung bean [29], highlighting potential genotype-dependent responses.

Association analysis revealed significant positive associations between seed Mg and P concentrations under NS conditions, consistent with findings in other legumes, such as soybean, pea, lentil, and beans [51], and sesame (*Sesamum indicum* L.) [52]. A strong positive associationalso occurred between seed protein and S concentrations, as reported in chickpea [53,54]. Conversely, seed Ca and K concentrations negatively correlated, consistent with the results in sesame [52].

Significant positive correlations were observed under HS between seed P and Zn concentrations, and between seed protein and Mg concentrations, consistent with previous studies on chickpea [55], winter oilseed rape [56], and soybean [57]. A positive correlation between seed Zn and S concentrations was also noted, aligning with reports in wheat [58]. Conversely, significant negative correlations occurred under HS for seed Ca and K concentrations, and for seed Fe and Cu concentrations. While the negative correlation between seed Ca and K is in agreement with findings in sesame [52], the negative correlation between seed Fe and Cu diverges from a reported positive correlation in the same sesame study.

Clustering analysis grouped the genotypes into two distinct clusters under NS and HS conditions. Distant genotypes within each cluster could be valuable for breeding programs aimed at developing nutrient-rich, heat-tolerant chickpea varieties. Similar clustering-based selection strategies have been successfully applied in chickpea [59], lentil [60], and common bean [61]. It is thought that the chickpea genotypes used in this study have different heat stress thresholds, as in Phaseolus species having different heat stress tolerances or sensitivities [62], which affect the variation of some macro and micronutrient contents.

## Conclusions

Heat stress at 35/20°C significantly reduced chickpea yield and adversely affected seed quality, including decreased C, Ca, Cu and Fe concentrations. However, seed protein and P, K, S, Mg, Mn, and Zn concentrations were either less affected or increased under HS. The positive correlations observed between seed protein and both Mg and P suggest that enhancing protein levels could also improve other nutritional traits under HS. Therefore, maintaining an optimal balance of seed C, protein, and other essential nutrients could help meet chickpea’s calorific and nutritional demandsas temperatures continue to rise. Future research focusing on the molecular mechanism underlying the reductions in C, Ca, Mg, and Fe concentrations under HS could provide valuable insights into improving these nutrient contents in chickpea under HS conditions. Chickpeas with higher nutritional values under HS conditions could be potentially used in breeding programs to improve chickpea seed nutritional quality.

## Supporting information

S1 File S1 Fig. Growth chamber temperatures recorded during chickpea growth. S1 Table Chickpea (*Cicer arietinum* L.) accessions used in the study. S2 Table Combined/ interaction effect of eight chickpea genotypes under non stress and heat stress on seed nutrition components.
(DOCX)

## References

[1] Jha UC, Nayyar H, Thudi M, Beena R, Vara Prasad PV, Siddique KHM. Unlocking the nutritional potential of chickpea: strategies for biofortification and enhanced multinutrient quality. Front Plant Sci. 2024;15:1391496. doi: 10.3389/fpls.2024.1391496 38911976 PMC11190093

[2] Food and Agriculture Organization of the United Nations S division (FAO). FAOSTAT. FAOSTAT Database. http://www.fao.org/faostat/en/#data/QC Accessed 2023

[3] Kaushal N, Awasthi R, Gupta K, Gaur P, Siddique KHM, Nayyar H. Heat-stress-induced reproductive failures in chickpea (Cicer arietinum) are associated with impaired sucrose metabolism in leaves and anthers. Funct Plant Biol. 2013;40(12):1334–49. doi: 10.1071/FP13082 32481199

[4] Kalra N, Chakraborty D, Sharma A, Rai HK, Jolly M, Chander S, et al. Effect of increasing temperature on yield of some winter crops in northwest India. Curr Sci. 2008;:82–8.

[5] Devasirvatham V, Gaur PM, Mallikarjuna N, Raju TN, Trethowan RM, Tan DKY. Reproductive biology of chickpea response to heat stress in the field is associated with the performance in controlled environments. Field Crops Res. 2013;142:9–19. doi: 10.1016/j.fcr.2012.11.011

[6] Jha UC, Kumar Y, Katiyar PK. Capturing genetic variability in chickpea (Cicer arietinum L.) germplasm, released varieties, and advanced breeding lines under heat stress environment in North Indian climatic condition. Indian J Genet Plant Breed. 2023;83(02):270–2. doi: 10.31742/isgpb.83.2.14

[7] Jha UC, Bohra A, Parida SK, Jha R. Integrated “omics” approaches to sustain global productivity of major grain legumes under heat stress. Plant Breed. 2017;136(4):437–59. doi: 10.1111/pbr.12489

[8] Rani A, Devi P, Jha UC, Sharma KD, Siddique KHM, Nayyar H. Developing Climate-Resilient Chickpea Involving Physiological and Molecular Approaches With a Focus on Temperature and Drought Stresses. Front Plant Sci. 2020;10:1759. doi: 10.3389/fpls.2019.01759 32161601 PMC7052492

[9] Jeffrey C, Trethowan R, Kaiser B. Chickpea tolerance to temperature stress: Status and opportunity for improvement. J Plant Physiol. 2021;267:153555. doi: 10.1016/j.jplph.2021.153555 34739858

[10] Bhandari K, Sita K, Sehgal A, Bhardwaj A, Gaur P, Kumar S, et al. Differential heat sensitivity of two cool‐season legumes, chickpea and lentil, at the reproductive stage, is associated with responses in pollen function, photosynthetic ability and oxidative damage. J Agron Crop Sci. 2020;206(6):734–58. doi: 10.1111/jac.12433

[11] Devi P, Jha UC, Prakash V, Kumar S, Parida SK, Paul PJ, et al. Response of Physiological, Reproductive Function and Yield Traits in Cultivated Chickpea (Cicer arietinum L.) Under Heat Stress. Front Plant Sci. 2022;13:880519. doi: 10.3389/fpls.2022.880519 35720547 PMC9202580

[12] Summerfield RJ, Hadley P, Roberts EH, Minchin FR, Rawsthorne SJEA. Sensitivity of chickpeas (Cicer arietinum) to hot temperatures during the reproductive period. Expt Agric. 1984;20(1):77–93.

[13] Wang J, Gan YT, Clarke F, McDonald CL. Response of Chickpea Yield to High Temperature Stress during Reproductive Development. Crop Sci. 2006;46(5):2171–8. doi: 10.2135/cropsci2006.02.0092

[14] Devasirvatham V, Gaur PM, Raju TN, Trethowan RM, Tan DKY. Field response of chickpea (Cicer arietinum L.) to high temperature. Field Crops Res. 2015;172:59–71. doi: 10.1016/j.fcr.2014.11.017

[15] Upadhyaya HD, Dronavalli N, Gowda CLL, Singh S. Identification and Evaluation of Chickpea Germplasm for Tolerance to Heat Stress. Crop Sci. 2011;51(5):2079–94. doi: 10.2135/cropsci2011.01.0018

[16] Pattison AL, Uddin MN, Trethowan RM. Use of in-situ field chambers to quantify the influence of heat stress in chickpea (Cicer arientinum). Field Crops Res. 2021;270:108215. doi: 10.1016/j.fcr.2021.108215

[17] Benali A, El Haddad N, Patil SB, Goyal A, Hejjaoui K, El Baouchi A, et al. Impact of Terminal Heat and Combined Heat-Drought Stress on Plant Growth, Yield, Grain Size, and Nutritional Quality in Chickpea (Cicer arietinum L.). Plants (Basel). 2023;12(21):3726. doi: 10.3390/plants12213726 37960082 PMC10650860

[18] Devi P, Awasthi R, Jha U, Sharma KD, Prasad PVV, Siddique KHM, et al. Understanding the effect of heat stress during seed filling on nutritional composition and seed yield in chickpea (Cicer arietinum L.). Sci Rep. 2023;13(1):15450. doi: 10.1038/s41598-023-42586-0 37723187 PMC10507029

[19] Jha UC, Priya M, Naik YD, Nayyar H, Thudi M, Punnuri SM, et al. Major abiotic stresses on quality parameters in grain legumes: impacts and various strategies for improving quality traits. Environ Expt Bot. 2024;2024:105978.

[20] Kumar S, Bhushan B, Wakchaure GC, Dutta R, Jat BS, Meena KK, et al. Unveiling the impact of heat stress on seed biochemical composition of major cereal crops: Implications for crop resilience and nutritional value. Plant Stress. 2023;9:100183. doi: 10.1016/j.stress.2023.100183

[21] Sehgal A, Sita K, Bhandari K, Kumar S, Kumar J, Vara Prasad PV, et al. Influence of drought and heat stress, applied independently or in combination during seed development, on qualitative and quantitative aspects of seeds of lentil (Lens culinaris Medikus) genotypes, differing in drought sensitivity. Plant Cell Environ. 2019;42(1):198–211. doi: 10.1111/pce.13328 29744880

[22] Choukri H, El Haddad N, Aloui K, Hejjaoui K, El-Baouchi A, Smouni A, et al. Effect of High Temperature Stress During the Reproductive Stage on Grain Yield and Nutritional Quality of Lentil (Lens culinaris Medikus). Front Nutr. 2022;9:857469. doi: 10.3389/fnut.2022.857469 35495922 PMC9051399

[23] Sita K, Sehgal A, Bhandari K, Kumar J, Kumar S, Singh S, et al. Impact of heat stress during seed filling on seed quality and seed yield in lentil (Lens culinaris Medikus) genotypes. J Sci Food Agric. 2018;98(13):5134–41. doi: 10.1002/jsfa.9054 29635707

[24] Samineni S, Mahendrakar MD, Hotti A, Chand U, Rathore A, Gaur PM. Impact of heat and drought stresses on grain nutrient content in chickpea: Genome-wide marker-trait associations for protein, Fe and Zn. Environ Expt Bot. 2022;194:104688.

[25] Jha UC, Shafi S, Tallury S, Nayyar H, Udgata AR, Ciampitti IA, et al. Dynamic changes in seed nutritional components of mung bean [(Vigna radiata (L.) R. Wilczek)] under heat stress. Sci Rep. 2025;15(1):12586. doi: 10.1038/s41598-025-93992-5 40221429 PMC11993610

[26] Gieseking JE, Snider HJ, Getz CA. Destruction of Organic Matter in Plant Material by the Use of Nitric and Perchloric Acids. Ind Eng Chem Anal Ed. 1935;7(3):185–6. doi: 10.1021/ac50095a021

[27] Kjeldahl J. Neue Methode zur Bestimmung des Stickstoffs in organischen Körpern. Fresenius, Zeitschrift f anal Chemie. 1883;22(1):366–82. doi: 10.1007/bf01338151

[28] Aloui K, Choukri H, El Haddad N, Gupta P, El Bouhmadi K, Emmrich PMF, et al. Impact of Heat and Drought Stress on Grasspea and Its Wild Relatives. Plants (Basel). 2023;12(19):3501. doi: 10.3390/plants12193501 37836241 PMC10574926

[29] Priya M, Bhardwaj A, Jha UC, HanumanthaRao B, Prasad PVV, Sharma KD, et al. Investigating the influence of elevated temperature on nutritional and yield characteristics of mung bean (Vigna radiata L.) genotypes during seed filling in a controlled environment. Front Plant Sci. 2023;14:1233954. doi: 10.3389/fpls.2023.1233954 37810386 PMC10551632

[30] Yang H, Gu X, Ding M, Lu W, Lu D. Heat stress during grain filling affects activities of enzymes involved in grain protein and starch synthesis in waxy maize. Sci Rep. 2018;8(1):15665. doi: 10.1038/s41598-018-33644-z 30353095 PMC6199321

[31] Ahmed N, Tetlow IJ, Nawaz S, Iqbal A, Mubin M, Nawaz ul Rehman MS, et al. Effect of high temperature on grain filling period, yield, amylose content and activity of starch biosynthesis enzymes in endosperm of basmati rice. J Sci Food Agric. 2015;95(11):2237–43. doi: 10.1002/jsfa.6941 25284759

[32] Liu P, Guo W, Jiang Z, Pu H, Feng C, Zhu X, et al. Effects of high temperature after anthesis on starch granules in grains of wheat (Triticum aestivum L.). J Agric Sci. 2011;149(2):159–69. doi: 10.1017/S0021859610001024 22505772 PMC3320814

[33] Castro M, Peterson CJ, Rizza MD, Dellavalle PD, Vázquez D, Ibanez V, et al. Influence of heat stress on wheat grain characteristics and protein molecular weight distribution. Wheat Production in Stressed Environments: Proceedings of the 7th International Wheat Conference. Springer Netherlands. 2007. p. 365–71.

[34] Tanaka H, Gorafi YSA, Fujita M, Sasaki H, Tahir ISA, Tsujimoto H. Expression of seed storage proteins responsible for maintaining kernel traits and wheat flour quality in common wheat under heat stress conditions. Breed Sci. 2021;71(2):184–92. doi: 10.1270/jsbbs.20080 34377066 PMC8329878

[35] Wang J, Yuan B, Xu Y, Huang B. Differential responses of amino acids and soluble proteins to heat stress associated with genetic variations in heat tolerance for hard fescue. J Am Soc Hort Sci. 2018;143(1):45–55.

[36] Krishnan HB, Kim W-S, Oehrle NW, Smith JR, Gillman JD. Effect of Heat Stress on Seed Protein Composition and Ultrastructure of Protein Storage Vacuoles in the Cotyledonary Parenchyma Cells of Soybean Genotypes That Are Either Tolerant or Sensitive to Elevated Temperatures. Int J Mol Sci. 2020;21(13):4775. doi: 10.3390/ijms21134775 32635665 PMC7370294

[37] Batra D, Dhull SB, Rani J, Meenakshi M, Kumar Y, Kinabo J. Effect of heat stress on seed protein quality in mungbean [Vigna radiata (L.) Wilczek]. Legume Sci. 2023;5(4). doi: 10.1002/leg3.205

[38] Nayyar H, Kaur S, Singh S, Upadhyaya HD. Differential sensitivity of Desi (small‐seeded) and Kabuli (large‐seeded) chickpea genotypes to water stress during seed filling: effects on accumulation of seed reserves and yield. J Sci Food Agric. 2006;86(13):2076–82. doi: 10.1002/jsfa.2574

[39] Laira MD, Andrade SA, Silveira NM, Machado EC, R Ribeiro RV, Zambrosi FC. High post-flowering phosphorus status promotes the tolerance of soybean to terminal heat stress. Environ Expt Bot. 2023;215:105501.

[40] Lin L, Wu J, Jiang M, Wang Y. Plant Mitogen-Activated Protein Kinase Cascades in Environmental Stresses. Int J Mol Sci. 2021;22(4):1543. doi: 10.3390/ijms22041543 33546499 PMC7913722

[41] Hermans C, Verbruggen N. Physiological characterization of Mg deficiency in Arabidopsis thaliana. J Exp Bot. 2005;56(418):2153–61. doi: 10.1093/jxb/eri215 15983014

[42] Kang X, Zhao L, Liu X. Calcium Signaling and the Response to Heat Shock in Crop Plants. Int J Mol Sci. 2023;25(1):324. doi: 10.3390/ijms25010324 38203495 PMC10778685

[43] Heckathorn SA, Downs CA, Sharkey TD, Coleman JS. The small, methionine-rich chloroplast heat-shock protein protects photosystem II electron transport during heat stress. Plant Physiol. 1998;116(1):439–44. doi: 10.1104/pp.116.1.439 9449851 PMC35186

[44] Noctor G, Foyer CH. ASCORBATE AND GLUTATHIONE: Keeping Active Oxygen Under Control. Annu Rev Plant Physiol Plant Mol Biol. 1998;49:249–79. doi: 10.1146/annurev.arplant.49.1.249 15012235

[45] Kocsy G, Szalai G, Galiba G. Effect of heat stress on glutathione biosynthesis in wheat. Acta Biologica Szegediensis. 2002;46(3–4):71–2.

[46] Ali MM, Waleed Shafique M, Gull S, Afzal Naveed W, Javed T, Yousef AF, et al. Alleviation of Heat Stress in Tomato by Exogenous Application of Sulfur. Horticulturae. 2021;7(2):21. doi: 10.3390/horticulturae7020021

[47] El Haddad N, En-Nahli Y, Choukri H, Aloui K, Mentag R, El-Baouchi A, et al. Metabolic Mechanisms Underlying Heat and Drought Tolerance in Lentil Accessions: Implications for Stress Tolerance Breeding. Plants (Basel). 2023;12(23):3962. doi: 10.3390/plants12233962 38068599 PMC10708188

[48] Ullah A, Romdhane L, Rehman A, Farooq M. Adequate zinc nutrition improves the tolerance against drought and heat stresses in chickpea. Plant Physiol Biochem. 2019;143:11–8. doi: 10.1016/j.plaphy.2019.08.020 31473401

[49] Shiraya T, Mori T, Maruyama T, Sasaki M, Takamatsu T, Oikawa K, et al. Golgi/plastid-type manganese superoxide dismutase involved in heat-stress tolerance during grain filling of rice. Plant Biotechnol J. 2015;13(9):1251–63. doi: 10.1111/pbi.12314 25586098 PMC6680209

[50] Ye Y, Medina-Velo IA, Cota-Ruiz K, Moreno-Olivas F, Gardea-Torresdey JL. Can abiotic stresses in plants be alleviated by manganese nanoparticles or compounds?. Ecotoxicol Environ Saf. 2019;184:109671. doi: 10.1016/j.ecoenv.2019.109671 31539809

[51] Grembecka M, Szefer P. Elemental Profiles of Legumes and Seeds in View of Chemometric Approach. Appl Sci. 2022;12(3):1577. doi: 10.3390/app12031577

[52] Teboul N, Gadri Y, Berkovich Z, Reifen R, Peleg Z. Genetic Architecture Underpinning Yield Components and Seed Mineral-Nutrients in Sesame. Genes (Basel). 2020;11(10):1221. doi: 10.3390/genes11101221 33081010 PMC7603122

[53] Sandhu SS, Keim WF, Hodges HF, Nyquist WE. Inheritance of Protein and Sulfur Content in Seeds of Chickpeas. Crop Sci. 1974;14(5):649–52.

[54] Mondal SS, Mandal P, Saha M, Bag A, Nayak S, Sounda G. Effect of potassium and sulphur on the productivity, nutrient uptake and quality improvement of chickpea. J Crop Weed. 2005;1(2):84–6.

[55] Vandemark GJ, Grusak MA, McGee RJ. Mineral concentrations of chickpea and lentil cultivars and breeding lines grown in the U.S. Pacific Northwest. Crop J. 2018;6(3):253–62. doi: 10.1016/j.cj.2017.12.003

[56] Eifler J, Wick JE, Steingrobe B, Möllers C. Genetic variation of seed phosphorus concentration in winter oilseed rape and development of a NIRS calibration. Euphytica. 2021;217(4). doi: 10.1007/s10681-021-02782-3

[57] Webb JR, Ohlrogge AJ, Barber SA. The effect of magnesium upon the growth and the phosphorus content of soybean plants. Soil Sci Soc Am J. 1954;18(4):458–62.

[58] Şule Orman. Effects of sulphur and zinc applications on growth and nutrition of bread wheat in calcareous clay loam soil. Afr J Biotechnol. 2012;11(13). doi: 10.5897/ajb11.2701

[59] Srungarapu R, Mohammad LA, Mahendrakar MD, Chand U, Jagarlamudi Venkata R, Kondamudi KP, et al. Genetic variation for grain protein, Fe and Zn content traits in chickpea reference set. J Food Composition and Analysis. 2022;114:104774. doi: 10.1016/j.jfca.2022.104774

[60] Bansal R, Bana RS, Dikshit HK, Srivastava H, Priya S, Kumar S, et al. Seed nutritional quality in lentil (Lens culinaris) under different moisture regimes. Front Nutr. 2023;10:1141040. doi: 10.3389/fnut.2023.1141040 37396135 PMC10313473

[61] Murube E, Beleggia R, Pacetti D, Nartea A, Frascarelli G, Lanzavecchia G, et al. Characterization of Nutritional Quality Traits of a Common Bean Germplasm Collection. Foods. 2021;10(7):1572. doi: 10.3390/foods10071572 34359442 PMC8306501

[62] Eker T, Sari H, Sari D, Canci H, Arslan M, Aydinoglu B, et al. Advantage of Multiple Pods and Compound Leaf in Kabuli Chickpea under Heat Stress Conditions. Agronomy. 2022;12(3):557. doi: 10.3390/agronomy12030557

