# Supplementary material for: Influence of elevated temperature on the nutritional profile of Chickpea (Cicer arietinum L.) Seeds
Source: PLoS One. 2025 Aug 22;20(8):e0330230. doi: 10.1371/journal.pone.0330230 (PMC12373242; doi:10.1371/journal.pone.0330230)
Supplement: S1 File — S1 Fig. Growth chamber temperatures recorded during chickpea growth. S1 Table Chickpea (Cicer arietinum L.) accessions used in the study. S2 Table Combined/ interaction effect of eight chickpea genotypes under non stress and heat stress on seed nutrition components. (DOCX) [file pone.0330230.s001.docx]

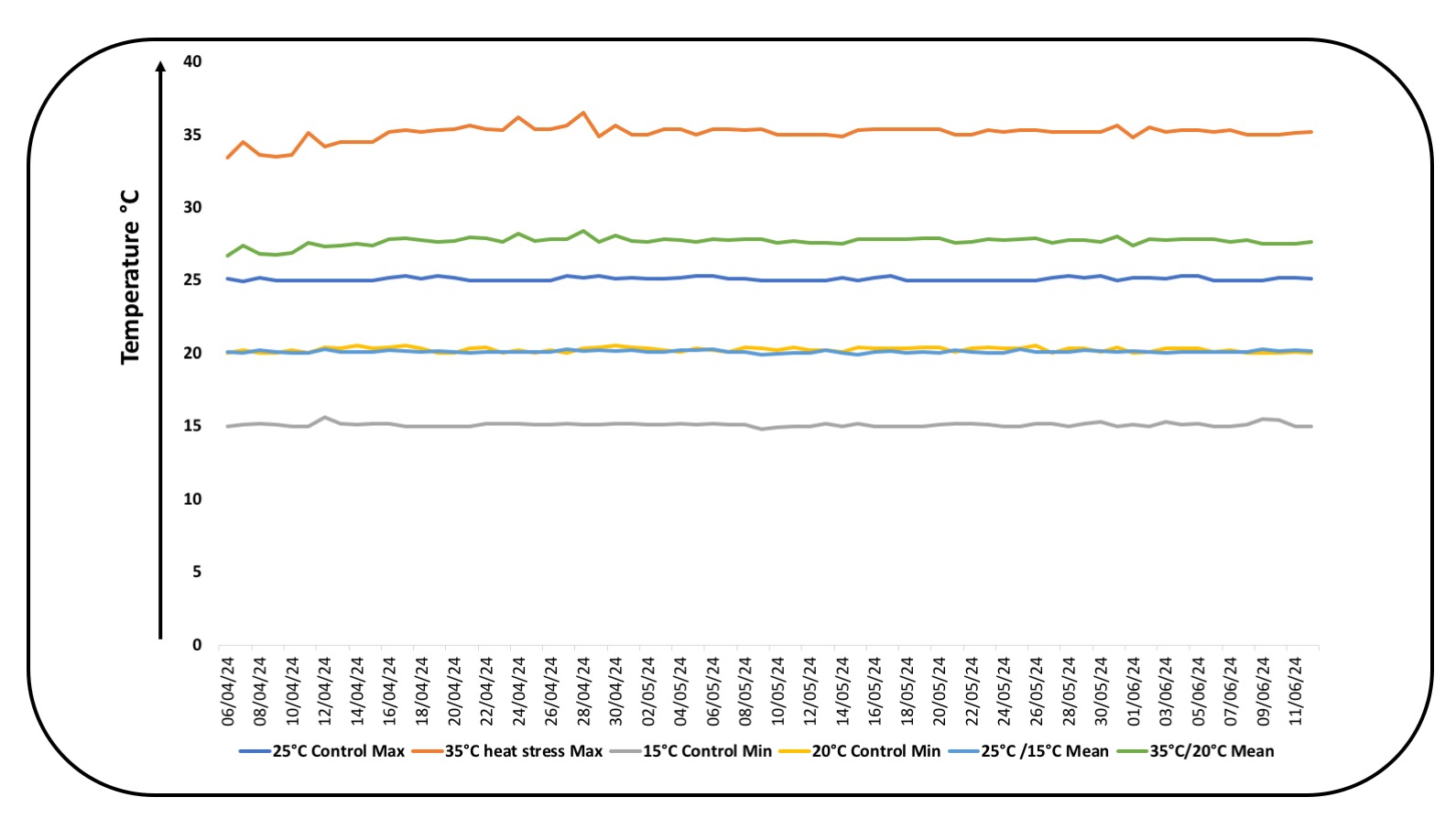


**S1 Fig**. Growth chamber temperatures recorded during chickpea growth.

| **S1 Table Chickpea (*Cicer arietinum* L.) accessions used in the study.** | | | |
| --- | --- | --- | --- |
| **ACCESSION** | **NAME** | **ORIGIN** |  |
| PI 372596 (Desi) | RPIP12-069-0519 | India |  |
| PI 360688 (Kabuli) | RIP12-153-05499 | Philippines |  |
| PI 368485 (Kabuli) | Krnjveski | North Macedonia |  |
| PI 598080 (Desi) | Myles | USA, Washington |  |
| PI 513144 (Desi) | Chana | Pakistan |  |
| PI 360691(Desi) | RPIP12-155-00848 | Egypt |  |
| PI 518255(Desi) | Tyson | Australia |  |
| Gokce (Kabuli) | – | Turkey |  |

|  | **C%** |  | **Protein%** |  | **P %** |  | **K %** |  | **Ca %** |  | **Mg %** |  |
| --- | --- | --- | --- | --- | --- | --- | --- | --- | --- | --- | --- | --- |
|  | **NS** | **HS** | **NS** | **HS** | **NS** | **HS** | **NS** | **HS** | **NS** | **HS** | **NS** | **HS** |
| **PI368485** | 41.2^a^ | 40.58^cd^ | 26.4^a^ | 22^bc^ | 0.5^cde^ | 0.649^a^ | 1.39^abc^ | 1.55^a^ | 0.065^e^ | 0.091^cde^ | 0.162^efg^ | 0.203^a^ |
| **PI360688** | 41.12^ab^ | 41.18^ab^ | 17.83^def^ | 16.3^def^ | 0.408^gh^ | 0.441^fg^ | 1.40^abc^ | 1.51^ab^ | 0.088^cde^ | 0.093^cde^ | 0.145^h^ | 0.154^gh^ |
| **Gokce** | 41.15^ab^ | 41.12^ab^ | 15.73^ef^ | 18.9^cde^ | 0.381^h^ | 0.548^bc^ | 1.22^c^ | 1.51^ab^ | 0.152^ab^ | 0.103^cd^ | 0.159^fg^ | 0.183^bcd^ |
| **PI360691** | 41.11^ab^ | 40.44^cd^ | 21.96^bc^ | 22.83^b^ | 0.505^bcde^ | 0.555^b^ | 1.39^abc^ | 1.28^c^ | 0.102^cde^ | 0.123^bc^ | 0.192^ab^ | 0.186^bc^ |
| **PI518255** | 40.73^bc^ | 40.19^d^ | 17 ^def^ | 17.1^def^ | 0.498^cde^ | 0.545^bc^ | 1.30^c^ | 1.40^abc^ | 0.161^a^ | 0.141^ab^ | 0.164^efg^ | 0.174^cde^ |
| **PI513144** | 40.72^bc^ | 40.42^cd^ | 15.43^ef^ | 19.83^bcd^ | 0.547^bc^ | 0.536^bcd^ | 1.57^a^ | 1.55^ab^ | 0.077^de^ | 0.084^de^ | 0.189^b^ | 0.170^ef^ |
| **PI598080** | 40.69^bc^ | 40.58^cd^ | 22.9^b^ | 16.4^def^ | 0.482^ef^ | 0.489^def^ | 1.35^bc^ | 1.35^bc^ | 0.151^ab^ | 0.164^a^ | 0.169^ef^ | 0.168^ef^ |
| **PI372596** | 40.54^cd^ | 40.12^d^ | 14.2^f^ | 16.93^def^ | 0.467^ef^ | 0.547^bc^ | 1.21^c^ | 1.30^c^ | 0.168^a^ | 0.137^ab^ | 0.172^de^ | 0.184^bcd^ |

**S2 Table Combined/ interaction effect of eight chickpea genotypes under non stress and heat stress on seed nutrition components**

(Duncan's multiple range test (DMRT) with a significance level of p < 0.05)

S2 Table continued...

|  | **S %** |  | **Cu (ppm)** |  | **Fe (ppm)** |  | **Mn (ppm)** |  | **Zn (ppm)** |  | **SYP(g)** |  |
| --- | --- | --- | --- | --- | --- | --- | --- | --- | --- | --- | --- | --- |
|  | **NS** | **HS** | **NS** | **HS** | **NS** | **HS** | **NS** | **HS** | **NS** | **HS** | **NS** | **HS** |
| **PI368485** | 0.225^def^ | 0.299^a^ | 2.36^b^ | 2.23^b^ | 58.83^cd^ | 68.43^cd^ | 26.5^f^ | 37.4^cd^ | 70.26^b^ | 91.43^a^ | 7.56^b^ | 2.56^i^ |
| **PI360688** | 0.221^ef^ | 0.247^cde^ | 2.13^b^ | 1.83^b^ | 97.7^ab^ | 68.43^cd^ | 33^cdef^ | 26.9^f^ | 78.86^ab^ | 69.03^b^ | 5.16^cd^ | 2.71^i^ |
| **Gokce** | 0.222^ef^ | 0.28^abc^ | 4.96^a^ | 1.73^b^ | 63.16^cd^ | 77.7^abc^ | 25.16^f^ | 30.43^def^ | 67.66^b^ | 84.3^ab^ | 5.36^c^ | 3.39^g^ |
| **PI360691** | 0.252^bcde^ | 0.258^bcd^ | 3.2^b^ | 3.2^b^ | 80.8^abc^ | 41.53^d^ | 26.43^f^ | 27.23^ef^ | 69.63^b^ | 69.16^b^ | 4.1^e^ | 2.9^hi^ |
| **PI518255** | 0.228^def^ | 0.286^ab^ | 2.56^b^ | 2.5^b^ | 65.33^cd^ | 52.66^cd^ | 45.66^ab^ | 41.06^bc^ | 76.13^ab^ | 69.4^b^ | 8.76^a^ | 4.9^d^ |
| **PI513144** | 0.235^def^ | 0.295^a^ | 2.46^b^ | 2.76^b^ | 59.23^cd^ | 59.1^cd^ | 31.6^def^ | 35.83^cde^ | 83.63^ab^ | 81.26^ab^ | 7.46^b^ | 3.9^ef^ |
| **PI598080** | 0.249^cde^ | 0.239^def^ | 2.76^b^ | 2.56^b^ | 101.1^a^ | 58.13^cd^ | 38.3^bcd^ | 52.76^a^ | 83.03^ab^ | 67.9^b^ | 7.8^b^ | 3.53^fg^ |
| **PI372596** | 0.208^f^ | 0.259^bcd^ | 2.4^b^ | 2.13^b^ | 81.3^abc^ | 69.76^bcd^ | 37.13^cd^ | 28.53^ef^ | 70.16^b^ | 72.96^b^ | 7.8^b^ | 3.22^gh^ |

NS=on-stress, HS=heat stress
