# Supplementary material for: Influence of elevated temperature on the nutritional profile of Chickpea (Cicer arietinum L.) Seeds
Source: PLoS One. 2025 Aug 22;20(8):e0330230. doi: 10.1371/journal.pone.0330230 (PMC12373242; doi:10.1371/journal.pone.0330230)
Supplement: Attachment — Submitted filename: PONE-D-25-29542-Editor.docx [file pone.0330230.s002.docx]

Dear Editor,

I have reviewed the manuscript (PONE-D-25-29542) "**Influence of Elevated Temperature on the Nutritional Profile of Chickpea (*Cicer arietinum* L.) Seeds**". My comments and suggestions are provided below, along with some details.

I have searched the following keywords, including “chickpea”, “heat”, and “nutrition”, in the Web of Science (WoS) and found a limited number of studies on the effects of heat stress on the nutritional profile of chickpeas.

I made considerable corrections and comments on the manuscript. Mn can be considered for possible publication after revision. Author(s) should avoid self-citation. Also, author(s) should use line numbers to easy review the mn.

Best regards,

Dr. Cengiz TOKER
