# Supplementary material for: Influence of elevated temperature on the nutritional profile of Chickpea (Cicer arietinum L.) Seeds
Source: PLoS One. 2025 Aug 22;20(8):e0330230. doi: 10.1371/journal.pone.0330230 (PMC12373242; doi:10.1371/journal.pone.0330230)
Supplement: Attachment — Submitted filename: PONE-D-25-29542-Author.docx [file pone.0330230.s003.docx]

Dear author(s),

I have reviewed yor manuscript (PONE-D-25-29542) "**Influence of Elevated Temperature on the Nutritional Profile of Chickpea (*Cicer arietinum* L.) Seeds**". My comments and suggestions are provided below:

Title

1. Seeds in the title can be removed since seeds are used for nutrition in chickpea. Alternative title: **Influence of Heat Stress on the Nutritional Profile of Chickpea (*Cicer arietinum* L.)**

Abstract

2. Change “those of chickpea” to “nutritional profile of chickpea”
3. A blank between effect and of
4. Change “cultivating eight chickpea genotypes” to “eight cultivated chickpea genotypes”
5. The following sentence must not be italicized *seed carbon (C, %), protein (%), phosphorus (P, %), potassium (K, %), magnesium (Mg, %), sulfur (S, %),* and *manganese (Mn, ppm)*
6. Change “seed (S%), seed iron (Fe, ppm),” to “sulfur (S%), iron (Fe, ppm),”. Readers are already aware that the edible portion of cultivated chickpeas is the seed.
7. Change “seed copper (Cu, ppm),” to “copper (Cu, ppm),”.
8. Change “seed C” to “C”
9. Change “seed M” to “Mg”
10. Change “seed protein” to “protein”
11. Please check others.

Introduction

12. Before reference [2] (BR2), FAOSTAT data should be updated, and the 2023 data (available). Write in 2023 in the sentence for readers.
13. BR3, The reference 3 should be written properly. Please avoid self-citation.
14. BR3-6, India is the largest chickpea-producing country; however, the given samples on heat stress (˃32°C) may be representative of a broader context (in the world).
15. BR12, Read the articles below for more and different references: <https://doi.org/10.3390/agronomy12030557>; <https://doi.org/10.1038/s41598-022-05559-3>.
16. BR13-14, a blank between that and temperatures
17. BR15, a blank between yield and by
18. BR17-19, a blank before [17-19]
19. BR20, a blank at the end of the sentence.
20. BR19, a blank before [19]
21. BR24, a blank before [24]
22. BR18, a blank between with and heat-sensitive
23. A blank between nutrition and traits
24. A blank between into and developing
25. Please use more references on chickpea.

M&M

Plant materials

26. Check Gockce? Why did you use one cultivar?

Experimental setup

27. What was the irrigation quantity?
28. A blank before non-stress
29. Some traits of the soil used in the experiment should be detailed.
30. Sowing, planting and harvesting dates should be given.
31. Change “Data analysis” to Data analyses”. There was more than one analysis.
32. Duncan or Tukey tests could be used instead of LSD.

Results

33. Remove soil from “Under normal soil conditions,” because NS was explained as non-stress (NS) conditions.
34. Remove the seed as well. You explained seed analyses before for readers.
35. Write conditions after NS
36. Change “under high salinity (HS) conditions” to “under heat stress (HS) conditions”
37. Check and correct seed yield per plant (SYP) under heat-stress (HS) conditions! 34.9 is higher than NS.

Protein and carbon concentration

38. Change “decreased protein concentrations” to “a decrease in protein concentrations”
39. Change “increased seed C” to “an increase in seed C”
40. Remove “seed” because you mentioned that analyses were made in the seed in M&M.

Micronutrients

41. Change “Gockce” to “Gokce”.

Seed yield per plant

42. Remove “identifying it as a potential candidate for heat tolerance breeding” from here to “Discussion”.
43. Abbreviations (G, T, HS, NS etc) were repeated. Please insist on full name or abbreviations through text.

Discussion

44. BR37, a blank before [37].
45. A blank between to and HS
46. BR39, a blank before has.
47. The scientific name of mung bean can be written.
48. The common names of *Lentil* and *Lathyrus* should be given.
49. The following sentence could be written as reference [63] “It is thought that the chickpea genotypes used in this study have different heat stress thresholds, as in *Phaseolus* species having different heat stress tolerances or sensitivities [63], which affect the variation of some macro and micronutrient contents.” 63.	Tene et al., Star of biochemical traits of heat-tolerant and heat-sensitive *Phaseolus* genotypes in coping with heat stress. Plant Growth Regulation. 2025; doi: <https://doi.org/10.1007/s10725-025-01340-4>

Conclusion

50. The following sentence or similar sentence could be added in Conclusion, too. Chickpeas having the higher nutritional values under HS conditions were suggested to use in breeding programs.

References

51. All references should be checked and corrected according to “Guide for Authors”.
52. The scientific names of plant species should be italicized.

I hope you will find as useful my suggestion to improve your mn. Please use line numbers. Review was taken more time than I thought due to without line no.
